# Immersive Virtual Reality Simulation in Forensic Psychiatry and Adjacent Clinical Fields: A Review of Current Assessment and Treatment Methods for Practitioners

**DOI:** 10.3389/fpsyt.2021.673089

**Published:** 2021-05-28

**Authors:** Kristina Sygel, Märta Wallinius

**Affiliations:** ^1^Department of Psychiatry and Neurochemistry, Centre for Ethics, Law and Mental Health, Institute of Neuroscience and Physiology, The Sahlgrenska Academy at University of Gothenburg, Gothenburg, Sweden; ^2^Department of Forensic Psychiatry, National Board of Forensic Medicine, Stockholm, Sweden; ^3^Research and Development Unit, Regional Forensic Psychiatric Clinic, Växjö, Sweden; ^4^Lund Clinical Research on Externalizing and Developmental Psychopathology, Child and Adolescent Psychiatry, Department of Clinical Sciences Lund, Lund University, Lund, Sweden

**Keywords:** forensic psychiatry, virtual reality, offenders, mental disorders, assessment, treatment

## Abstract

**Background:** Research has indicated that interactive, computerized case simulations using immersive virtual reality (VR) technology may be beneficial in the augmentation of conventional methods of assessment and treatment in forensic psychiatry, primarily through providing an engaging and safe environment in which the user can practice and learn skills and behaviors. However, there does not appear to be an overview of current developments available in the field, which may be an obstacle to clinicians considering the use of VR in their clinical practice.

**Objectives:** Current, clinically relevant assessment and treatment methods applying immersive VR in forensic or adjacent clinical settings, were analyzed.

**Methods:** This review surveyed the practical use of immersive VR in forensic psychiatry and relevant adjacent psychiatric and forensic fields from 2016 to 2020 and was performed in accordance with the Preferred Reporting Items for Systematic Reviews and Meta-Analyses (PRISMA) guidelines.

**Results:** Out of the 1,105 journal articles screened, 14 met criteria for inclusion. Four articles described VR interventions directly addressing forensic psychiatric settings (treatment of general aggression and assessment of sexual offenders against children). The majority of the remaining articles were in the clinical domain of psychosis treatment. Several interventions were designed as part of comprehensive treatment programs, and others were intended as one-off assessments or paired with pre-existing psychological treatment. The degree to which the VR simulations were individualized to the user appeared to be largely dependent upon the extent of provider input. A variety of research methodologies were used in the included articles and the majority had limitations common to small-scale, non-randomized studies. None of the studies reported serious adverse effects.

**Discussion:** There is a lack of large randomized controlled trials of current assessments or treatments using VR simulation in forensic psychiatry, let alone those with long-term follow-up, showing clear advantages of VR over standard practice. The evidence thus far is insufficient to recommend immediate and large-scale implementation of any one VR intervention, however, several have been shown to be feasible and acceptable to the participants and to provide insights and inspiration for future research and development.

## Introduction

Forensic psychiatry (FP) can be defined as the interface between psychiatry and criminal law. FP research often involves areas such as assessment of legal insanity, recidivism risk, and of treatment methods meant to reduce reoffending among individuals with criminal behavior and concomitant psychopathology (1, 2). Individuals assessed or treated within FP settings often suffer from multiple mental disorders of varying severity, in combination with psychosocial difficulties and criminal behavior. Despite international differences as to the severity or type of mental disorder required to access FP services, there are similarities in FP patient characteristics between jurisdictions. The Swedish national FP register includes 86% of all FP patients in the country. It reports the following primary ICD-10 (3) diagnoses as being the most common among FP patients: schizophrenia and other psychotic disorders (~67% of patients), neuropsychiatric disorders (~11%), personality disorders, affective disorders, intellectual disability (each ~5%) and substance use disorders (~3%). Several types of disorders are much more common as secondary than as primary diagnoses. For instance, over 80% of FP patients receive some form of substance use treatment during their stay in FP care (4). Swedish FP patients can be compared to a sample of long-stay patients in English medium and high security FP clinics in which the most prevalent diagnosis was found to be schizophrenia, followed by personality disorders (antisocial and borderline being the most common), intellectual disability and substance abuse issues (5). Similarly, an Australian study of not guilty by reason of mental illness forensic patients over 25 years found that the main primary diagnosis recorded for the patient sample was schizophrenia-spectrum disorder, and the most common co-morbid disorders were personality disorders, substance abuse disorders, and intellectual disability (6).

Despite being a combined subset of psychiatric patients and criminal offenders, the assessment and treatment of FP patients cannot simply be extrapolated from general psychiatry or programs within prison and probation services. For example, FP patients frequently have poor literacy skills (7), low motivation for assessment and treatment (8), suffer from multiple psychiatric disorders (6, 9), and are involuntarily restricted in their interactions with their community. Unfortunately, for former FP patients and for society at large, reoffending and readmission are common after discharge (10), and increase with length of follow-up (11, 12). Internationally it has been recognized that there is a need for evidence-based methods for both assessment and treatment specifically within FP (1, 2, 8, 13, 14). A recent review of the state of the art of research within FP care demonstrated serious knowledge gaps in all areas identified as important for FP treatment, including diagnostic and risk assessments, psychological interventions and rehabilitation (15). Thus, there is an urgent need for further research in the field in order to provide evidence-based methods to rehabilitate FP patients while reducing their risk of reoffending, and this research could potentially benefit from recent technological developments within the so-called eHealth field (16).

Virtual reality (VR) can be defined as a real-time computer simulated environment experienced using several sensory modalities (such as via a head-mounted display goggles and headphones) thus creating a sense of being present in the artificial environment. In a review paper on clinical VR, Rizzo et al. (17) argue that, during the last two decades, VR has transitioned from expensive entertainment into a technology capable of delivering previously unachievable psychiatric assessment and treatment opportunities. The authors state that a key feature of clinical VR is in its ability to generate dynamic yet controlled multisensory interactive simulations, as well as register and analyze the user's behavioral responses. These properties can be harnessed to promote a number of processes in psychiatric/psychological treatment for example, exposure to a feared or coveted stimulus, motivation to perform otherwise boring repetitive tasks, measurement of cognitive performance, and facilitating participation through engaging attention. A higher level of immersion into a virtual environment appears to improve the user's sense of being present and his/her emotional engagement (18). This may, for example, be achieved through using immersive VR as opposed to two-dimensional computer simulation, There is an expanding body of literature on the use of clinical VR in general psychiatry, particularly in the areas of post-traumatic stress disorder, anxiety and phobias, schizophrenia, eating disorders, substance related disorders, and chronic pain rehabilitation (19, 20). Several authors have called for the FP field to join its ranks (8, 16).

Interactive, non-immersive, computerized case simulations have previously been explored in the augmentation of conventional methods of assessment and treatment of violent offenders, and research has cautiously indicated that such programs may be beneficial (8, 21, 22). However, Ticknor (23), recently proposed that VR simulation (including semi-immersive and non-immersive types) can be a “game changer” in American correctional rehabilitation. VR may facilitate treatment and reintegration of offenders into society through treatment of psychiatric disorders (e.g., using VR cognitive behavioral therapy), teaching necessary skills, providing feedback, and making virtual treatment groups possible. In their systematic review of technological interventions to improve the treatment of forensic psychiatric patients, Kip et al. (8) found only two articles describing interventions using VR simulation in forensic settings up until December 2017. Both articles presented cross-sectional quantitative studies aimed at validating the use of VR simulations as stimuli in the assessment and/or treatment of sexual offenders and incorporated physiological measures such as penile plethysmography (24, 25). In a study interviewing Dutch FP therapists and patients, Kip et al. (16) explored in detail how VR may be of added value in forensic mental health in the future. VR was postulated to be particularly suited to the forensic context given the limitations of the FP setting (e.g., involuntary incarceration, threat to safety of others), complex patient population (e.g., heterogenous psychopathology and offending behavior, low levels of literacy and motivation for treatment) and type of treatments required. The main areas in which VR was postulated to be most useful were in patient skills training, providing the opportunity for the patients to observe situations or stimuli, and in revealing patient behaviors/characteristics to treatment providers. However, several barriers remain to the implementation of VR in FP settings and, in order to overcome these, a review of the recent, rapid progress in VR in FP and adjacent clinical settings is necessary.

## AIMS

Through a systematic literature review of experiments published in international journals from 2016 onwards, our aim was to survey the current use of immersive VR in forensic psychiatry and relevant adjacent psychiatric and forensic fields, with a view to identifying and analyzing promising new, present-day assessment and treatment methods which may eventually be adapted to, and potentially improve, FP practice. This study is a part of an extensive research program, FORevidence, providing evidence-based interventions for use within the Swedish FP care setting (grant no. 2018-01409) with the ultimate purpose of increasing mental health while reducing recidivism and thus benefiting both the individual patient and society as a whole. As its ultimate purpose is to be of current practical use in developing VR interventions for FP, emphasis has been placed upon identifying and examining the ongoing work of relevant clinical VR projects, programs and research groups around the world, rather than on creating a comprehensive theoretical overview of clinical VR field and its development. It is hoped that the results of this study may guide FP clinicians as to the types of VR interventions currently being used or developed by research groups and may even inspire international collaborations.

## Methods

The systematic review was performed in accordance with the Preferred Reporting Items for Systematic Reviews and Meta-Analyses, PRISMA guidelines (26).

### Inclusion and Exclusion Criteria

Peer reviewed articles detailing quantitative, experimental studies of immersive clinical VR technology used/proposed for use for assessment or treatment in adult FP and relevant adjacent psychiatric and forensic fields were included. As it was predicted that few randomized controlled trials (RCTs) addressing this type of intervention in this target population would be available, non-randomized studies of interventions were also included. In line with the objective of this systematic review, to find current uses of VR technology which can be adapted to FP conditions, only original studies published in English from 2016 and onwards were included. Review articles were excluded as they were deemed not to include sufficient details of original experiments or protocols. Non-immersive computer simulations, e.g., those presented on a two-dimensional screen, or articles in which the type of technology used was ambiguous, were also excluded. Due to the very small number of results generated in the forensic domain, the searches conducted on VR in FP and VR and offenders were performed without time limit and vetted by hand. From the search results generated on VR in psychiatry/mental disorders, the specific areas deemed to be of greatest relevance to FP were selected based on the most common primary diagnoses in the Swedish National Quality Registry for Forensic Psychiatry (4) i.e., schizophrenia and other psychotic disorders, neuropsychiatric disorders, personality disorders, affective disorders (bipolar I and schizoaffective disorder), and substance abuse disorders, as well as intellectual disability. This selection of relevant diagnoses is deemed to apply to FP populations in several other countries, as described above, despite differences between jurisdictions. Thus, popular general psychiatric applications of VR, such as treatment of anxiety, phobias, eating disorders, post-traumatic stress disorder, were excluded in favor of the diagnostic areas listed above. Articles in which VR was used as a tool to further the understanding of the condition itself, rather than directly for assessment or treatment, were also excluded, as they were deemed too far removed from clinical implementation in the near future.

### Literature Search

Three on-line electronic literature searches were carried out in each of the following three databases: PubMed, PsycINFO and Web of Science for the time period 1 January 2016–31 August 2020. The three searches were performed in each database in the following domains: VR and forensic psychiatry, VR and offenders, VR and mental disorders. In PubMed, the searches were performed using MeSH terms and “all fields” searches for the following terms: Virtual reality AND forensic psychiatry OR forensic mental health; Virtual reality AND crime OR criminal OR prisons OR prisoners; Virtual reality AND crim^*^ OR prison^*^ OR offender^*^ OR probation; Virtual reality AND mental disorder. In PsycINFO, searches were performed using the following “exploded” thesaurus terms in “anywhere”: Virtual reality AND forensic psychiatry; virtual reality AND criminal offenders OR prisoners OR prisons OR probation; virtual reality AND mental disorder OR psychiatry. In Web of Science searches, the search term virtual reality was used in conjunction with the following truncated terms: forensic^*^; criminal^*^ or offender^*^ or prison^*^; psych^*^ or mental^*^. In the majority of the searches, the exclusion criteria were applied manually at the level of the title or abstract screening process. In the searches in which more than 300 entries were found, filters (journal article, full text available, English, adults, specific time frame, humans) were also applied directly to the electronic search before beginning the manual screening process. In the Web of science VR and mental disorders search, irrelevant subject areas (e.g., surgery) were excluded using the “visualization treemap” before the title screening. The main author performed all the literature searches. To verify the search methodology, the searches in PubMed and PsycINFO were compared with those performed by an experienced librarian at Region Kronoberg, using the same search terms.

As described above, the search criteria were widely set so as to miss as few studies as possible due to for example indexing according to symptoms (e.g., delusions or aggression) or setting (e.g., correctional services) rather than population or diagnostic category. The main author subsequently manually vetted all the search results according to the inclusion and exclusion criteria in all but one of the nine searches. The Web of Science search within the psychiatry/psychology domain generated a disproportionately large number of results (3 764 results) with many sub-domains obviously irrelevant to the study (e.g., virtual reality in surgery, environmental science, ophthalmology). These were removed by electronically excluding such subject groups from the display of search results before commencing the manual vetting process. During the manual vetting of all the search results, the majority of articles excluded were discarded based on their title. If a title did not provide sufficient information for or against inclusion, the article abstract was examined. Subsequently, duplicate articles were removed and the vetted articles read in their entirety. The articles still found to be matching the inclusion criteria were then included.

### Data Extraction

The results were presented in two tables, one for VR and FP and the other for VR in diagnostic areas related to FP. The area of clinical application of the VR simulation (VRS), goal of the VRS, type of study, conclusion of the study and advantages and disadvantages of the VRS and study were presented, in accordance with the inclusion of non-randomized studies in systematic reviews described in the Cochrane Handbook of Systematic reviews (27).

## Results

**Figure 1:**
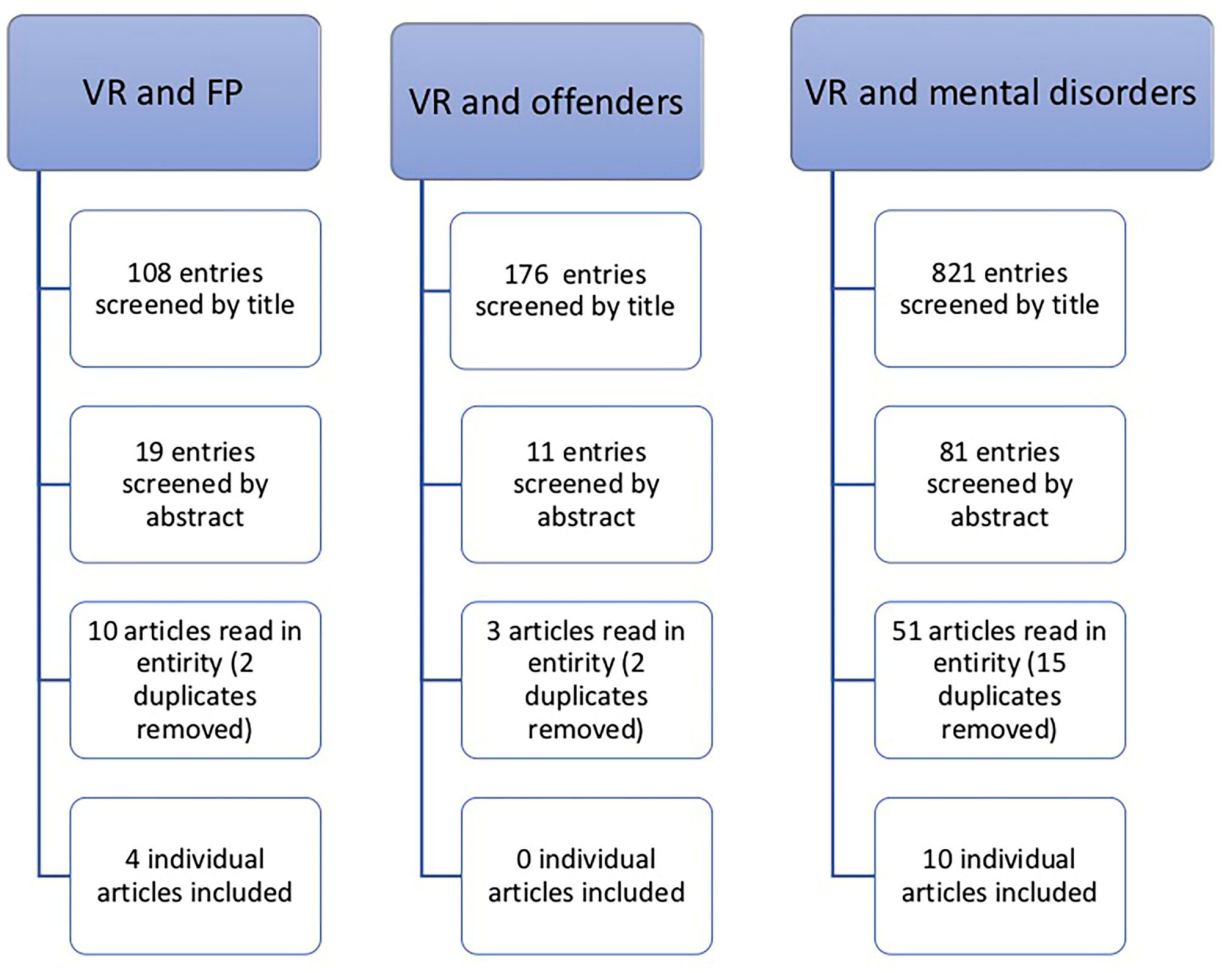
Aggregated results of the searches performed in PubMed, PsycINFO, and Web of Science databases.

The search strategy and number of articles included are shown in Figure 1. The main reason for excluding results during the manual vetting process were that they did not meet inclusion criteria due to lack of relevance to the study topic, primarily that non-immersive VR technology had been used, and that the subject matter did not pertain to psychiatric/psychological interventions (e.g., remote heart monitoring or computer visualization in forensic pathology). Other common reasons were that the results were purely theoretical or review articles and thus did not provide sufficient experimental detail for the purpose of the current study.

In Tables 1, 2, the included studies are provided together with factors deemed likely to be advantages or disadvantages of potential implementation of the individual VR interventions in FP, first with regard to articles found in the domain of VR and offender populations (FP and correctional services), and subsequently those found in the VR and psychiatry/psychology category.

**Table 1:** VR and forensic psychiatry.

**Clinical application of VR simulation (VRS)**	**Name of VRS, reference, country**	**Goal of VRS**	**Type of study of VRS**	**Conclusion of study**	**Salient advantages/disadvantages of VRS and study**
Assessment of sexual offenders against children (SOCs)	Fromberger et al. (28), Germany	Behavioral monitoring of SOCs through virtual risk situations e.g., meeting a child while shopping in a supermarket, in addition to assessment as usual	Non-blinded NRSI feasibility study in FP SOCs vs. non-offender controls; self-report/rating & therapist ratings (*n =* 13)	VR risk situations provided additional information for risk management; showed good tolerability & ecological validity	*Advantages*: Stimuli individualized though prior participant ratings; meticulous VR reconstruction of environment, control group *Disadvantages*: limited choices of responses in the VRS; small NRSI, no test of predictive validity
Treatment of general aggression	VRAPT, (29), Netherlands	VR Aggression Prevention Therapy (VRAPT); manualized, CBT-based intervention to reduce reactive aggressive behavior of FP patients; 16 sessions	Study protocol of single blinded multicenter RCT [see (30), below]	N/AAim of RCT will be to investigate the effectiveness of the VRAPT (n ~ 128)	*Predicted advantages*: stand-alone treatment; individualized stimuli (through manualized interaction with provider); physical measures of arousal included; single blind RCT design *Predicted disadvantages:* no predicted limitations listed in study protocol
	VRAPT, (30), Netherlands	[See (29), above]	Non-blinded multicenter RCT in FP inpatients receiving TAU vs. waiting list controls; self-report & observational data; 12 wk follow up (*n =* 128)	No significant decrease in aggressive behavior; may temporarily influence on anger control skills, impulsivity & hostility	*Advantages*: [as for (29), above]; rigorous study design with an unusually large number of FP patients *Disadvantages:* provider training (16 h) required; provider interaction required during VRS; single blinding was not manitained
	VR-GAIME, (31), Netherlands	Virtual Reality Game for Aggressive Impulse Management; training avoidance movements to angry faces; 5 sessions	Study protocol of double blind multicenter RCT with placebo control in FP outpatients; in conjunction with Aggression Replacement Training (ART) (n~ 60)	N/A Aim of RCT will be to test the effects of VR-GAIME in outpatients with (predominantly) personality syndromes	*Advantages*: minimal provider training and participation required; double blind RCT design *Disadvantages*: VR-GAIME only given in conjunction with another treatment program; control game may also have positive effects; few treatment sessions planned; severe psychopathologies excluded from study (e.g., psychosis & bipolar disorder); few predicted limitations listed in study protocol

**Table 2:** VR in diagnostic areas related to forensic psychiatry.

**Clinical application of VR simulation (VRS)**	**Name of VRS, reference, country**	**Goal of VRS**	**Type of study of VRS**	**Conclusion of study**	**Salient advantages/disadvantages of VRS and study**
Assessment of alcohol abuse	Alco-VR (32), Spain	VR alcohol-cue environment used to differentiate between heavy and light drinkers	NRSI pilot study among college students (*n =* 25)	Choice of alcohol-cues in VRS more accurate at detecting heavy drinking than concomitant self-reported craving/anxiety	*Advantages*: Stand-alone assessment; limited provider interaction required *Disadvantages*: brief description of the VRS; small sample of mostly female college students without psychopathology
Assessment of psychosis (paranoid ideation)	Riches et al. (33), UK	Real-time assessment of paranoid ideation & associated social performance using a single 5 min bar-room scenario	NRSI pilot study (including some randomization) comparing high & low trait paranoia in healthy participants (*n =* 89)	Safe and effective for the assessment of people who experience high paranoid ideation in social situations	*Advantages*: minimal provider input required during VRS; a physical measure of arousal included *Disadvantages:* 1 short scenario; not individualized stimuli; evaluation of state paranoia pre and post VRS not during (e.g., eye gaze or HR); mostly female subjects with higher education, may have been underpowered
Treatment of psychosis (paranoid ideation and social avoidance)	Pot-kolder et al. (34), Netherlands	VR-based CBT for paranoid ideation & social avoidance; 16 sessions	Multi-center single-blind RCT among psychiatric outpatients, waiting list controls (*n =* 116)	Intervention significantly reduced momentary paranoid ideation & anxiety; No significant increase in amount of time spent with other people	*Advantages*: individualized avatars and provider interaction; data collection via electronic experience sampling; 6-month follow-up period; intervention delivered within standard psychiatric services; scientifically rigorous study design *Disadvantages*: limited conversational interaction in the VRS; intensive provider (CBT-trained psychologist) participation required; 2 days provider training required; 30% of eligible patients declined to participate/did not respond
Treatment of psychosis (delusions)	Dietrichkeit et al. (35), Germany	Ameliorate delusions through correcting cognitive distortions (overconfidence in memory); 2 intervention sessions	Case study from an ongoing RCT; 3 weeks follow up (*n =* 2)	Delusions and paranoia decreased; cybersickness must be addressed	*Advantages:* stand-alone intervention; RCT ongoing *Disadvantages:* cybersickness prevented the use of immersive VR in 1 of the 2 cases (seated); level of provider input required is unclear from article; case report study
Treatment of psychosis (auditory verbal hallucinations, AVH, in schizophrenia)	Dellazizzo et al. (36), Canada	VR avatar therapy (VRAT) for refractory AVH in treatment of resistant schizophrenia; 7 sessions	Case study; 3 months follow up (*n =* 1)	Severity of AVH, depressive symptoms, hostility and QOL improved	*Advantages*: stand-alone intervention, highly individualized stimuli through provider interaction, *Disadvantages*: not manualized thus requiring input from highly qualified provider; case report
	du Sert et al. (37), Canada	VRAT for refractory AVH in schizophrenia; 7 sessions [appears to be the same VRS as in (36)]	Non-blinded randomized partial cross-over pilot study with TAU as control condition (*n =* 19)	Significant improvements in AVH severity & related distress, depressive symptoms & QOL, lasting at 3-month follow-up	*Advantages*: stand-alone intervention; highly individualized stimuli through provider interaction; treatment resistant cases included *Disadvantages*: not manualized thus requiring input from “actor-like” provider (bilingual psychiatrist); 21% dropout due to anxiety & lack of engagement
Treatment of psychosis (cognitive rehabilitation in schizophrenia)	La Paglia et al. (38), Italy	VR in rehabilitation of cognitive deficits in schizophrenia (attention training tasks); 10 sessions	Non-blinded NRSI pilot study in psychiatric outpatients with “control” patients given psychological cognitive skills training (*n =* 15)	Both groups showed improvement in divided attention task but VRS group also showed improved general cognitive functioning, planning & sustained attention	*Advantages*: stand-alone intervention, manualized, limited provider interaction *Disadvantages*: no limitations listed in article, not individualized stimuli, study appears to include 2 experimental groups & no controls
Treatment of psychosis (social cognition and functioning)	DISCoVR (39), the Netherlands	Dynamic social cognition training in VR (DiSCoVR) for improving social cognition & social functioning in psychosis patients; 16 sessions	Study protocol of multicenter RCT with active control group (relaxation training VR)	Not applicable Aim of RCT will be to evaluate the efficacy of DISCoVR (n ~ 100)	*Predicted advantages*: stand-alone treatment; individualized stimuli through manualized interaction with provider; RCT planned *Predicted disadvantages:* simulation requires full provider participation; no limitations listed in study protocol
	DISCoVR, (40), the Netherlands	As Nijman et al. (39), above	Single-group feasibility and acceptability pilot study (*n =* 22)	Feasible and acceptable; significant improvement of emotion perception; no significant change in other measures of social cognition	*Advantages:* As for As Nijman et al. (39), above *Disadvantages:* intensive participation by qualified provider, uncontrolled pilot lacking in power, no standardized measures of feasibility & acceptability, possible selection of participants with less severe deficits
Treatment of Borderline personality disorder	Nararro-Haro et al. (41), USA	VR to facilitate mindfulness skills training in DBT; 4 sessions of mindfulness practice while in VR	Case study (*n =* 1)	Reduced urges to commit suicide, self-harm, quit therapy, use substances, as well as reduced negative emotions after each session	*Advantages*: minimal provider input during VR intervention; case with difficulties in practicing mindfulness chosen *Disadvantages*: Given in conjunction with pre-existing DBT; not individualized stimuli; case report

*AT, avatar therapy; AVH, auditory verbal hallucinations; CBT, cognitive behavioral therapy; DBT, dialectic behavioral therapy; FP, forensic psychiatry/psychiatric; NRSI, non-randomized study of intervention; QOL, quality of life; RCT, randomized controlled trial; SOC, sexual offenders against children; TAU, treatment as usual; VR, virtual reality; VRAT, VR avatar therapy; VRS, virtual reality simulation*.

## Discussion

This review provides an overview of recent (2016–2020) publications on assessment and treatment methods applying immersive VR in FP and relevant adjacent psychiatric and forensic fields, with the ultimate aim of identifying promising new methods which may eventually be adapted to, and potentially improve, clinical FP practice. The most extensively researched clinical domain pertaining to FP in which immersive VR has been used appears to be the treatment of psychosis, including individual psychotic symptoms such as paranoid delusions or auditory verbal hallucinations. In addition, interventions geared toward specific types of offenses (i.e., violence/aggression, sexual offenses against children) have been developed, implemented and evaluated. However, only one intervention was directed toward each of the large clinical FP domains of personality disorders and substance use disorders which clearly demonstrates the need for further developments.

Regarding treatment of psychosis, eight studies met the inclusion criteria for this review. Among them, an RCT of a 16 session VR-based cognitive behavioral therapy (CBT) program was found to significantly reduce momentary paranoid ideation and anxiety among psychiatric outpatients (34), a 16 session treatment program significantly improved emotion perception in patients with deficits in social cognition and functioning (40), and real-time VR assessment was found safe and effective for people who experience high levels paranoid ideation in social situations (33). Thus, there are methods available for implementation in FP settings which have demonstrated initial effects in patients with psychotic disorders. However, in-depth studies of the actual treatment mechanisms of these interventions are needed, and also studies comparing interventions using VR to interventions delivered without VR are required to determine the actual impact of the VR technology. The treatment interventions aimed at psychotic patients and included in this review were stand-alone treatments, with the majority being highly individualized and with provider interaction, thus requiring specific provider training. Even though many interventions were manualized, high demands were placed on the provider, an issue that needs to be considered in their clinical implementation.

Three of the studies included in this review used VR in broader behavioral, rather than diagnostic, areas such as general aggression, which are applicable to a large proportion of FP patients. Two of the studies were in study protocol form and presented future RCTs (29, 31). An RCT using the former protocol was completed within the time frame of this review and the intervention showed a temporary influence on anger control skills, impulsivity, and hostility, but no significant decrease in observed aggressive behaviors. Even though the number of studies is quite limited, they highlight important issues on how VR can be applied in treatments directed toward behavioral change (e.g., aggression), and not only specific symptoms such as paranoid delusions. More research is needed before any conclusions can be drawn. It would also be highly interesting to elucidate potential treatment moderators, to provide further knowledge on how to assess and evaluate behavioral change in FP settings.

Several of the studies identified within this review were smaller non-randomized studies of interventions (NSRIs) addressing the use of VR in clinical areas which are commonly excluded from other studies, such as treatment refractory schizophrenia (37) and severe borderline personality disorder (41). These patient groups are not particularly amenable to participation in clinical research, let alone large RCTs. Despite their modest sample sizes and mixed-methods, the pilot studies and case reports, such as those described above, are important and inspirational for treatment providers and VR developers. The above also illustrates the importance of expanding the horizon to adjacent and considerably larger clinical fields, such as general psychiatry, when scouting for interventions which may be adapted to FP.

As mentioned briefly above, several of the included studies have concerned VR-interventions used as part of more or less stand-alone treatments or assessments [e.g., (29, 30, 33, 39, 40)] others have been studied as adjuvant to other interventions, mainly other types of psychological therapy. In several studies it is indicated that patients have retained their ongoing pharmacological interventions. As patients often have on-going pharmacological and psychological treatments, including such patients in the studies makes the results easier to generalize to the entire FP population, possibly increasing ecological validity. However, a VR intervention that has been studied only when paired with a particular form of treatment is difficult to assess on its own. For example, in the case of the proposed study of VR-GAIME (31), the VR intervention is given in combination with Aggression replacement training (ART). Thus, its effect, and future clinical implementation, is difficult to disentangle from that of ART, a program which was discontinued by The Swedish Prison and Probation Service in 2010 due to unfavorable treatment outcome (42).

In most of the VR interventions described in this review, the degree to which the VRS can be individualized to the user appears to be dependent upon the extent of provider input, i.e., the interventions in which a trained therapist controls avatars in real-time appear to be more individualized than the more automated VR interventions requiring little provider input. This may have implications for reliability/generalizability of the study results but also for the implementation of the interventions in clinical practice. For instance, in the VR treatment studied by du Sert et al. (37), the provider is required to have “actor-like” qualities in order to tailor the VR treatment to the individual patient. As this is not a certified skill set traditionally sought when recruiting forensic psychiatric treatment providers, it is conceivable that this requirement limits the reliability and generalizability of the study results and of the VR treatment itself. In other VR programs, however, a high degree of attention has been paid to digitally individualizing the stimuli in a replicable way. For example, in the VR intervention described by Fromberger et al. (28), virtual child characters were selected based on the sexual offender's level of attraction to them and a digital supermarket with a plethora of groceries were painstakingly constructed. Despite this, the actual choices of interactions/behaviors available to the offender within the intervention appear to be limited to approaching or not approaching the virtual child character. Thus, the digital individualization and freedom of choice within the assessment/treatment programs studied do not appear to have been expanded and automated to their full potential in comparison with for example simulations in the gaming world (e.g., Lone Echo, www.oculus.com/lone-echo, or Half-life: Alyx, www.half-life.com/en/alyx, on Occulus Rift) or other cutting-edge advances in immersive virtual reality interventions for mental health (43). This appears to be yet another area to be considered when developing future VR interventions for clinical use in forensic settings.

In keeping with earlier reviews of VR in the FP field, such as by Kip et al. (8) and Ticknor (23), the evidence found in this review is insufficient to recommend any one particular immersive VR intervention as superior over the others. However, many interesting VR interventions that may be applicable to FP settings have been found, and more are being invented and studied by the day necessitating close monitoring of developments in the field. There is an obvious lack of properly powered RCTs of interventions using VRS, let alone those with long-term follow-up showing clear advantages of VR over standard practice in FP and FP-relevant contexts. To carry out such studies, multi-site and international collaborations are warranted and much needed in the field. Clearly, inspiration for this can be found in adjacent fields, the such as assessment and treatment of psychosis using VR.

A variety of research methodologies were used in the 14 articles presented in this review. Two were designed as single-blind RCTs, three as study protocols describing future RCTs, six were pilot studies with between 13 and 89 participants and the remaining three were case studies with one or two participants. Thus, the majority of the studies presented demonstrate limitations common to small-scale NRSIs such as risk of bias and limited validity, reliability and generalizability. However, none have reported any serious adverse effects such as worsening of symptomatology or increased aggression, which are important variables to consider in FP settings, indicating that the interventions may be used as inspiration or even a basis for future VR developments in FP.

Within clinical FP there are numerous areas in which the best evidence based practice has yet to be established such as psychological interventions and rehabilitation (15). While the FP research community is working toward closing these knowledge gaps through scientific enquiry, clinicians continue treating patients as they have always done i.e., adapting evidence gleaned from related clinical fields using their clinical experience, intuition and a sprinkling of entrepreneurship. The VR interventions described in this review may not as yet be scientifically proven to be either effective or ineffective. However, the theoretical arguments for why they could be particularly beneficial to FP patients are compelling and all the interventions have been found to be at the very least acceptable and harmless to the patients and their environment. Thus, despite the included studies not presenting high-grade, conclusive evidence in themselves, they are deemed to encourage further scientific and clinical exploration of VR as part of the on-going quest to discover and create safe and effective assessment and treatment methods in FP.

## Strengths and Limitations

A possible limitation of the current review is that all important and relevant studies may not have been included. This is likely to be due to this review having used broad subject search terms with a forensic psychiatric perspective such as “psychiatry” but studies possibly having been indexed on the level of the specific psychiatric symptoms for example “delusions” rather than by the types of illnesses in which such symptoms commonly are found. For example, “aggression” is a common symptom in FP but is no means a phenomenon limited to psychiatry or psychology or even to offenders. Individual types of offenses such as “intimate partner violence” may also have been indexed rather than the type of offender. The large disparity in number of entries found when performing the searches in the three databases suggests that indexing indeed is a problem in this field. Another limitation of this review is that it has not included other review-type articles, only original research. The excluded reviews may have mentioned other relevant original articles than those found using our search methods. The narrow time range studied in this review is a clear limitation as it is possible that research groups have published pilot studies several years ago and that larger studies are close to completion but as yet unpublished, particularly in the wake of the disruption that the 2020 COVID-19 pandemic has caused to large parts of society, including academia and healthcare. However, as we aim to provide an overview of the recent progress within this rapidly expanding field, shorter time spans between systematic reviews of VR interventions may be advantageous over the coming years.

## Conclusions

There is a clear lack of large clinical studies of current interventions using VR in FP settings. Incarcerated individuals with severe mental illness, often with a high risk of violence, are for practical and ethical reasons often difficult to enroll in scientific studies. Introducing any intervention in FP is a high stakes endeavor, as the outcome may significantly affect not only the future freedom and well-being of the individual patient but also the safety of the care staff and the community at large. This places heavy demands on the scientific validity of instruments intended for use within FP and on the rigor with which studies must be carried out in this population. In areas of psychiatry in which patients are more autonomous and accessible, studies are well under way, but there is still a lack of large, RCTs of stand-alone interventions showing clear advantages over treatment as usual. The urgent need for multi-site and international collaborations on this matter is obvious.

## Author Contributions

KS and MW designed the study and carried out the analysis/interpretation of the results. KS performed the literature search and wrote the first draft of the manuscript. MW wrote sections of the manuscript. Both authors contributed to manuscript revision, read, and approved the submitted version.

## Conflict of Interest

The authors declare that the research was conducted in the absence of any commercial or financial relationships that could be construed as a potential conflict of interest.
